# Wave‐driven rapid detachment of the skull in stranded sperm whales

**DOI:** 10.1002/ecy.70601

**Published:** 2026-11-12

**Authors:** Haruka Katada, Taro Okamura, Natsumi Mitsuoka, Mizuho Sano, Kaname Aihara, Naoko Miyakawa

**Affiliations:** ^1^ Graduate School of Environmental Studies Nagoya University Nagoya Aichi Japan; ^2^ School of Engineering Institute of Science Tokyo Tokyo Japan; ^3^ Graduate School of Natural Science and Technology Kanazawa University Kanazawa Ishikawa Japan; ^4^ Natural History Museum and Institute, Chiba Chiba Japan; ^5^ Present address: Sustainable System Research Laboratory Central Research Institute of Electric Power Industry Abiko Chiba Japan

**Keywords:** carrion ecology, cetacean strandings, decomposition, marine megafauna, necromass dispersal, *Physeter macrocephalus*, resource subsidies, taphonomy

Waves can rapidly destroy the carcasses of marine giants, thereby generating novel decompositional pathways. However, despite substantial ecological (e.g., Baptist et al., 2025; Moore et al., 2020; Quaggiotto et al., 2022) and paleontological interest (e.g., Bosio et al., 2021; Esperante et al., 2015; Reisdorf et al., 2012), the fate of stranded whales continuously exposed to natural wave action remains poorly understood. Existing knowledge has relied mainly on fragmentary carcasses with unknown postmortem intervals (e.g., Ramos et al., 2024) or on carcasses monitored after relocation to calm settings (e.g., Baptist et al., 2025). Fresh carcasses of large whales are often removed or dismantled shortly after stranding for public safety and management reasons (Geraci & Lounsbury, 2005; Tucker et al., 2018), leaving exceptionally few opportunities to monitor multiple individuals with known dates of death in situ over time.

On July 29, 2025, at approximately 18:00 JST, four young male sperm whales (*Physeter macrocephalus*), three of which measured 12.8–14.0 m in body length, were found live‐stranded at the mouth of the Sunomiya River on Heisaura Beach, Chiba, Japan (34°56′21″ N, 139°49′6″ E). The beach faces the open ocean and is exposed to high‐energy waves. All four whales were confirmed dead on July 31 (Day 0) and were left in situ without relocation or management‐related dismantling until burial on August 7 (Day 7). Individuals were designated A–D, and postmortem changes were assessed at 12 time points, generally around low tide, primarily using ground photographs. These records were supplemented by 3D scans and aerial drone observations conducted during the official scientific survey from August 5 to 7. Environmental conditions during the observation period were characterized using publicly available air temperature, relative humidity, and tide‐level data from the Japan Meteorological Agency (Japan Meteorological Agency, 2025a, 2025b). Offshore significant wave height ($H_{s}$, in meters) and energy mean wave period ($T_{m-1}$, in seconds; reported as “mean wave period”) were obtained from ERA5 (Copernicus Climate Change Service, Climate Data Store, 2023). Offshore wave power per unit crest length (P, in Watts per meter) was estimated from significant wave height ($H_{s}$) and energy mean wave period ($T_{m-1}$) using the deep‐water wave energy flux formulation (Guillou, 2020):

(1) $$P=\frac{\rho g^{2}}{64\pi }H_{s}^{2}T_{m-1}$$

where ρ and g are seawater density ($1025\;\mathrm{kg}\;m^{-3}$) and gravitational acceleration ($9.81\;m\;s^{-2}$), respectively (see Appendix S1 for details).

Three of the four whales (A, B, and D) showed detachment of the skeletonized skull and mandibles from the trunk within 1 week postmortem, and the fourth individual (C) developed dorsal neck constriction, indicating incipient detachment. The detached cranial skeletal remains were subsequently disarticulated into scattered elements, including the maxillae and occipital bones. Despite variation in decomposition rate, carcass decomposition followed a consistent sequence: (1) intact carcass (Figure 1a), (2) dorsal neck constriction, (3) detachment of the skeletonized skull and mandibles while cranial soft tissues remained attached to the trunk (Figure 1b,c), and (4) trunk cavity collapse (see https://doi.org/10.6084/m9.figshare.31145917.v1; Katada, 2026 and Appendix S1 for detailed chronological changes). These changes occurred under repeated wave exposure as the carcasses repeatedly stranded and refloated over successive tidal cycles. Offshore significant wave heights mostly exceeded 1.2 m, and estimated offshore wave power was generally around $10\;\mathrm{kW}\;m^{-1}$, with a brief increase above $40\;\mathrm{kW}\;m^{-1}$ during the early hours of August 2 (Day 2) (Figure 2).

**FIGURE 1:**
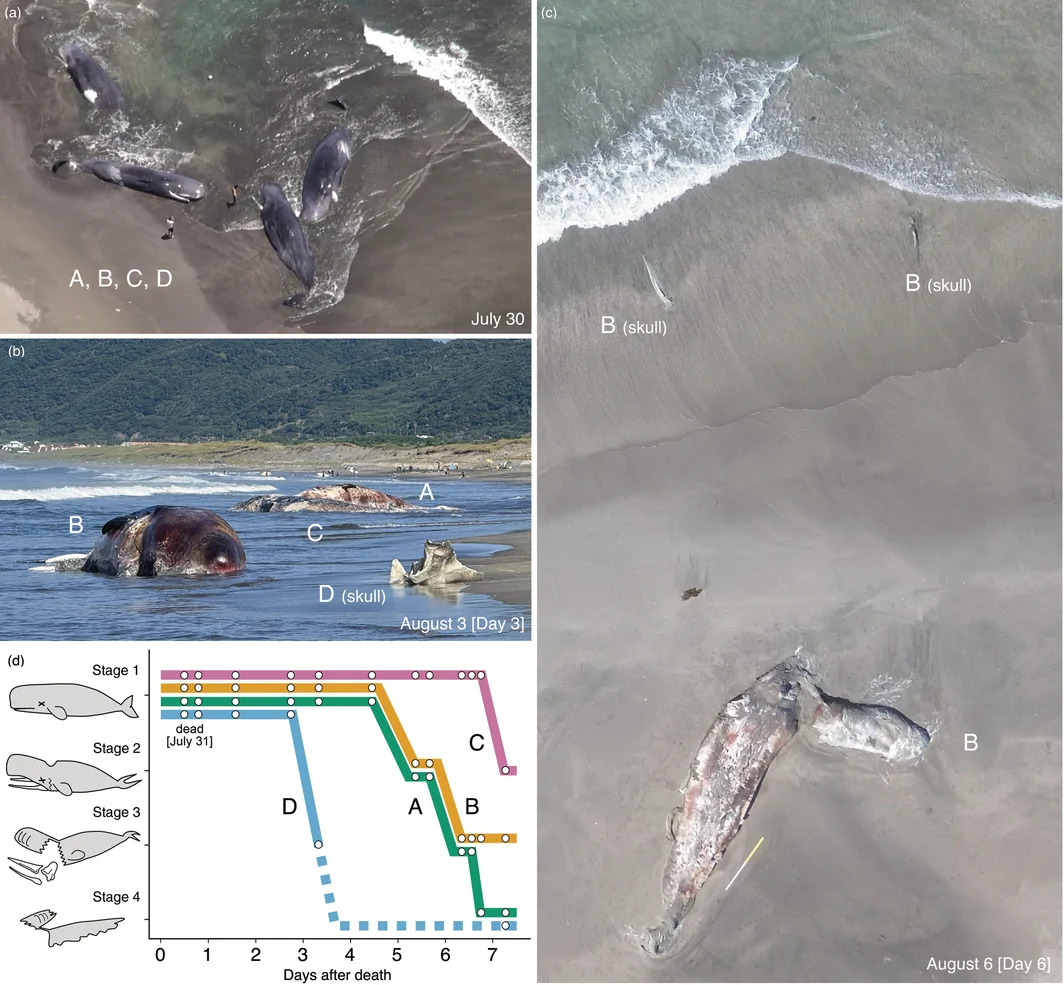
Wave‐driven decomposition of four stranded sperm whales on Heisaura Beach, Japan. (a) At least two of the four sperm whales were alive on July 30, one day after stranding; all four were confirmed dead on July 31, which was defined as Day 0. (b) On August 3 (Day 3), individuals A–C retained relatively intact body forms, whereas individual D had undergone rapid decomposition, with cranial skeletal elements detached from the trunk. (c) On August 6 (Day 6), cranial skeletal elements of individual B were detached from the trunk, while the cranial soft tissues remained attached to the trunk. (d) Chronological progression of decomposition in each individual, classified into four stages: Stage 1, intact carcass; Stage 2, dorsal neck constriction; Stage 3, detachment of the skeletonized skull and mandibles while the cranial soft tissues remain attached to the trunk; and Stage 4, trunk cavity collapse. Despite differences in decomposition rate and in the final stage documented for each individual, the observed changes in all individuals were consistent with this common sequence. Open circles indicate observation time points, and dashed lines indicate intervals during which the carcass was not directly observed. The image in panel (a) was provided by The Mainichi Newspapers Co., Ltd. Photographs in panels (b) and (c) were taken by Naoko Miyakawa and Natsumi Mitsuoka, respectively. Whale illustrations were created by Taro Okamura.

**FIGURE 2:**
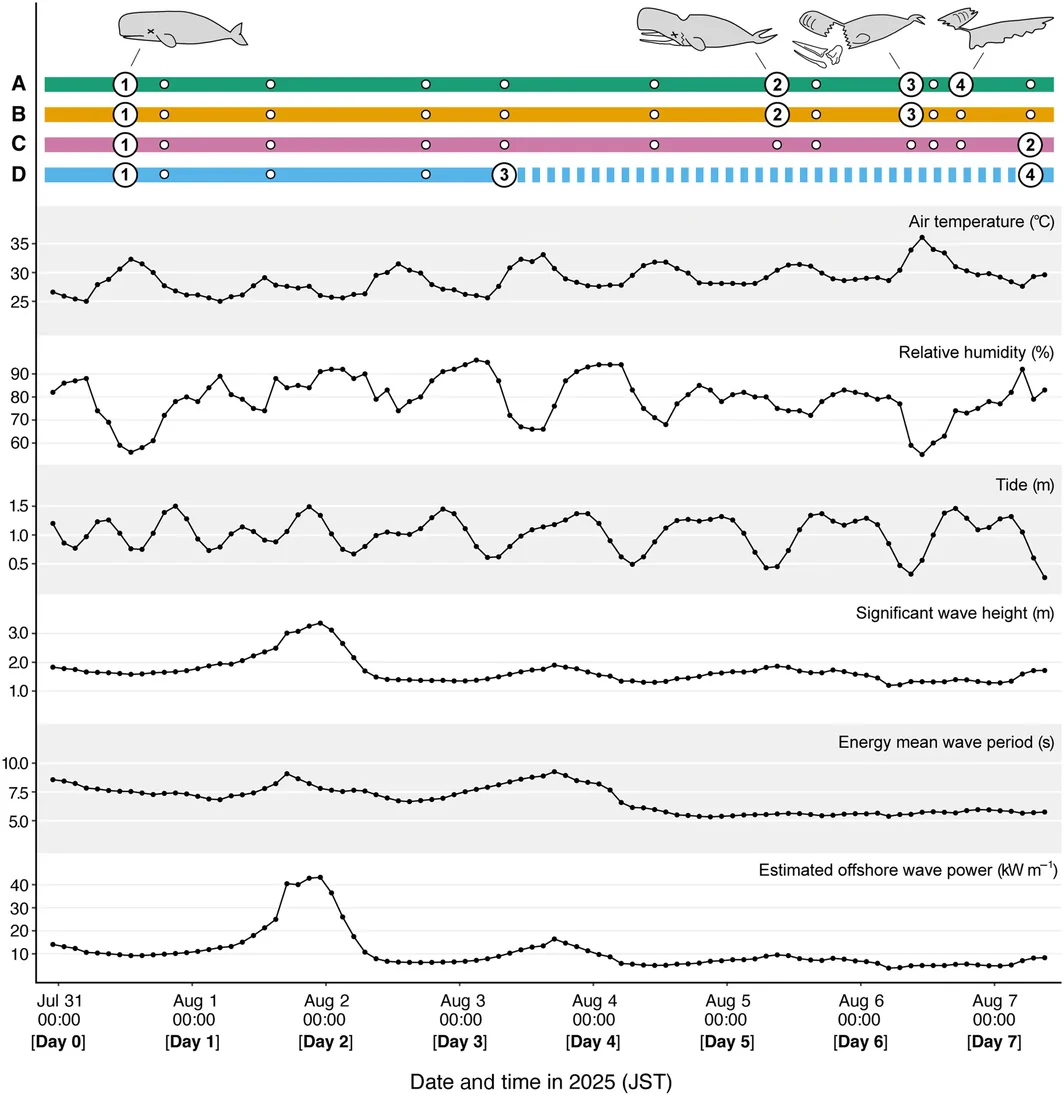
Temporal relationships between carcass decomposition and environmental conditions during the observation period. Dates and times are shown in Japan Standard Time (JST). The upper panel shows the progression of individuals A–D through the four decomposition stages defined in Figure 1d. Large numbered circles indicate the first documented occurrence of the corresponding stage, and small open circles indicate observation time points. Dashed lines indicate intervals during which the carcass was not directly observed. Whale illustrations depict the defining morphological features of each stage. The lower panels show air temperature, relative humidity, tide level, significant wave height, energy mean wave period, and estimated offshore wave power per unit crest length at 2‐h intervals. Offshore wave power was estimated from significant wave height and energy mean wave period using the deep‐water wave energy flux formulation described in the main text. Whale illustrations were created by Taro Okamura.

The observed sequence is broadly consistent with previous reports that the head region separates relatively early in floating carcasses (Okoshi, 2008; Schäfer, 1972). It also highlights the rapidity of this process in wave‐exposed stranded carcasses, with cranial skeletal detachment evident as early as 3 days postmortem in individual D. However, the observed sequence differed from that illustrated by Okoshi (2008) for sperm whales, in which the mandibles separate first, followed by detachment of the skull and cranial soft tissues from the trunk as a single unit. In the present case, the skull and mandibles detached from the trunk at approximately the same time, whereas the cranial soft tissues remained attached to the trunk. This asynchronous pattern likely resulted from early atlanto‐occipital dislocation caused by wave‐induced oscillations, while thick cervical adipose tissue retained the cranial soft tissues on the trunk side (Huggenberger et al., 2018).

All four whales stranded simultaneously on the same beach and thus experienced broadly similar large‐scale environmental conditions. Nevertheless, the timing of cranial skeletal detachment differed markedly among individuals and was likely driven by local differences in wave exposure within the stranding site (Figures 1d and 2). Individuals exposed to strong, continuous open sea waves (A and B) progressed more rapidly toward cranial skeletal detachment, whereas the individual stranded in a relatively sheltered area (C) progressed more slowly. Notably, individual D showed the most rapid change, with cranial skeletal detachment already evident by 3 days postmortem, while the other carcasses still retained relatively well‐preserved body forms at the same postmortem interval (Figure 1b). Although external damage from predation, fishing gear entanglement, or contact with artificial structures can affect decomposition rates, no obvious signs of such damage were observed in any individual (Baptist et al., 2025; Cassoff et al., 2011; Geraci & Lounsbury, 2005; Meynecke et al., 2024). Body lengths were similar among the three measured individuals (A–C: 12.8–14.0 m), and the recovered trunk of individual D exceeded 10 m in length, making body‐size differences unlikely to explain the observed variation. Together, these observations indicate that the timing of cranial skeletal separation can vary substantially even among carcasses within a single stranding site, underscoring the limits of inferring decomposition stage or stranding time from fragmentary remains.

Wave action likely promoted cranial skeletal detachment in the present case, but its effects should be interpreted in relation to other factors. Decomposition occurred under hot and humid summer conditions, with a mean air temperature of 28.7°C and a mean relative humidity of 78% during the study period (Figure 2). Such conditions likely enhanced microbial activity and putrefaction, thereby accelerating decomposition together with wave action (Rietz et al., 2025). By comparison, a previous Japanese case involved a sperm whale stranded in a sheltered bay during winter, where no cranial separation occurred even 2 months postmortem at a mean air temperature of approximately 17°C (Okabe et al., 2021). Age may also have influenced this process. In the present case, the detached cranial skeleton disarticulated into multiple elements, including the maxillae and occipital bones (Appendix S1: Figures S6, S8, S10, S11, and S13), suggesting that incomplete cranial fusion in young individuals may have facilitated skeletal separation. Therefore, the generality of rapid cranial skeletal detachment among wave‐exposed stranded sperm whales should be further examined in relation to temperature, age class, and degree of wave exposure.

Rapid cranial skeletal detachment under wave exposure indicates that stranded sperm whale carcasses can shift from whole‐carcass decomposition to separate decomposition pathways within days after death, generating distinct ecological and taphonomic outcomes. Ecologically, a skeletonized skull and a soft‐tissue‐covered trunk differ in tissue composition and nutritional properties (Clarke, 1979; Higgs et al., 2011), and may therefore function simultaneously as distinct coastal resources. In individual D, the separated cranial skeleton and trunk drifted approximately 1 km apart (Appendix S1: Figure S1), showing that carcass‐derived resources can become spatially dispersed within days. Taphonomically, early separation allows the skull and trunk to undergo independent transport and burial histories (Schäfer, 1972). Separation or loss of the cranial region despite preservation of a largely intact postcranial skeleton has been reported in extinct cetaceans (Esperante et al., 2015; Peters et al., 2009) and Mesozoic marine reptiles (Barnes & Hiller, 2010; Delsett et al., 2016). Although such occurrences span diverse depositional settings and cannot be attributed solely to the process documented here, our observations indicate that rapid postmortem physical disarticulation within days should be considered when interpreting these preservation patterns.

In conclusion, natural wave action can promote cranial skeletal detachment in stranded whale carcasses within days of death. Such rapid early‐stage decomposition may be overlooked in studies based on fragmentary carcasses with unknown postmortem intervals or on carcasses relocated to calmer settings. Future studies combining detailed field monitoring with experiments under controlled fluid conditions will further clarify the mechanical processes by which waves destroy whale carcasses.

## AUTHOR CONTRIBUTIONS

**Haruka Katada:** Conceptualization (lead); funding acquisition (lead); investigation (lead); writing—original draft (equal); writing—review and editing (equal). **Taro Okamura:** Funding acquisition (lead); investigation (equal); visualization (lead); writing—original draft (lead); writing—review and editing (equal). **Natsumi Mitsuoka:** Investigation (lead); visualization (lead); writing—original draft (equal); writing—review and editing (equal). **Mizuho Sano:** Investigation (equal); writing—review and editing (equal). **Kaname Aihara:** Investigation (equal); writing—review and editing (equal). **Naoko Miyakawa:** Investigation (lead); project administration (lead); writing—review and editing (equal).

## CONFLICT OF INTEREST STATEMENT

The authors declare no conflicts of interest.

## Supporting information


Appendix S1.

## Data Availability

Chronological drone and ground imagery, corresponding three‐dimensional datasets, and the environmental data used in this study (Katada, 2026) are available in Figshare at https://doi.org/10.6084/m9.figshare.31145917.v1.
