# Supplementary material for: Wave‐driven rapid detachment of the skull in stranded sperm whales
Source: Ecology. 2026 Nov 12;107(11):e70601. doi: 10.1002/ecy.70601 (PMC13756567; doi:10.1002/ecy.70601)
Supplement: Supplementary file 1 [file ECY-107-e70601-s001.pdf]

Appendix S1

Wave-driven rapid detachment of the skull in stranded sperm whales

Haruka Katada, Taro Okamura, Natsumi Mitsuoka, Mizuho Sano, Kaname Aihara, Naoko Miyakawa

Ecology

We documented the decomposition of four sperm whales that mass-stranded at Heisaura Beach, Tateyama City, Chiba, Japan, from the confirmation of their deaths on July 31, 2025, until their burial on August 7 (see main text). Previous large-whale strandings at this site involved only single individuals, making this the first recorded mass stranding of sperm whales at Heisaura Beach (Katooka et al., 2020; Sumi et al., 2022). The following sections describe data collection and chronological changes in decomposition, supported by photographs, drone footage, three-dimensional models, and environmental data. Figure S1 shows the site map, and Figures S2–S14 show daily photographs. All times are in Japan Standard Time (JST), and related datasets are available on Figshare (https://doi.org/10.6084/m9.figshare.31145917.v1; Katada 2026).

Data collection and carcass assessment

Multiple sources and methods were used to document temporal changes in carcass decomposition and position. Aerial footage recorded by helicopter at approximately 13:00 on July 30 was provided by Mainichi Newspapers Co., Ltd. and used with permission (main-text Figure 1a). Ground photographs from preliminary surveys conducted between July 31 and August 4 were similarly provided by the Awa Public Works Office and the Tateyama Marine Industries Office of the Chiba Prefectural Government. Additional photographs and videos were taken by N. Miyakawa (Natural History Museum and Institute, Chiba) on July 31 and August 3. During the official scientific survey from August 5 to 7, photographs were taken by H. Katada, N. Mitsuoka, T. Okamura, K. Aihara, M. Sano, and N. Miyakawa.

Carcass decomposition and position were assessed at 12 time points, generally around low tide, when wave action was relatively limited and the carcasses were stationary: July 31 at 12:00 and 19:00; August 1 at 14:00; August 2 at 18:00; August 3 at 08:00; August 4 at 11:00; August 5 at 09:00 and 16:00; August 6 at 09:00, 13:00 and 18:00; and August 7 at 06:30. Decomposition status was assessed from photographs, whereas carcass positions were estimated from GPS data and photographs. Based on the observed sequence of decomposition, carcass condition was classified into four stages: (1) intact carcass, (2) dorsal neck constriction, (3) detachment of the skeletonized skull and mandibles while cranial soft tissues remained attached to the trunk, and (4) trunk cavity collapse. These stage classifications are presented in the main text and Figures 1 and 2. Changes in carcass position from death to burial are shown in Figure S1c, and representative photographs documenting decomposition are presented in Figures S2–S14.

In addition, the condition of each sperm whale during early decomposition was recorded daily using the five codes defined by Geraci and Lounsbury (2005): Code I (alive), Code II (fresh carcass without bloating), Code III (moderate decomposition with bloating and skin sloughing), Code IV (advanced decomposition with severe bloating and loss of most epidermis), and Code V (skeleton exposed with detached skin). Because this classification does not necessarily represent a chronological progression of decay (Ramos et al., 2024), it was used only as a field-based description of carcass condition and was not interpreted as a sequence of terminal taphonomic stages.

Carcass movements while afloat during the official survey, including their responses to wave action, were documented through aerial drone surveys conducted by N. Mitsuoka using a DJI Mini 3 (SZ DJI Technology Co., Ltd.). Flights were conducted once on August 5 at 16:00, six times on August 6 at 08:00, 11:00, 14:00, 16:00, 17:00, and 18:00, and once on August 7 at 06:30. A planned flight during the morning low tide on August 5 was cancelled because of strong winds. The resulting drone footage was combined with ground photographs and videos to produce a single chronological video documenting changes in carcass condition and position.

Three-dimensional data were collected during morning low tides from August 5 to 7 by N. Mitsuoka, T. Okamura, and M. Sano using photogrammetry and LiDAR scanning. Photogrammetric models were generated from images captured with iPhone 15 Pro and iPhone 16 Pro devices (Apple Inc.) using Polycam (Polycam Inc.). LiDAR scans were obtained with an 11-inch iPad Pro (3rd generation, 2021; Apple Inc.) using Scaniverse (Niantic Inc.).

Environmental data and wave-power estimation

Environmental conditions during the observation period were characterized using meteorological, tidal, and wave data from publicly available datasets. Air temperature, relative humidity, and tide level were obtained at hourly resolution from databases maintained by the Japan Meteorological Agency (JMA; Japan Meteorological Agency, 2025a, b). Offshore wave conditions were obtained from the ERA5 reanalysis dataset (Copernicus Climate Change Service, Climate Data Store, 2023). Significant wave height (H_s; ERA5 variable "significant height of combined wind waves and swell," swh) and energy mean wave period (T_{m-1}; ERA5 variable "mean wave period," mwp) were extracted at 2-h intervals from the offshore ERA5 grid cell nearest to the study site (35.0° N, 140.0° E). ERA5 timestamps were converted from UTC to Japan Standard Time (JST; UTC+9) for comparison with field observations.

To provide an integrated measure of offshore wave conditions, wave power per unit crest length (P) was estimated from H_s and T_{m-1} using the deep-water wave energy flux formulation (Guillou, 2020):

$$P = \frac{\rho g^2}{64\pi} H_s^2 T_{m-1} \tag{S1}$$

where ρ is seawater density ($1025\ \mathrm{kg\,m^{-3}}$), g is gravitational acceleration ($9.81\ \mathrm{m\,s^{-2}}$), H_s is the significant height of combined wind waves and swell (m), and T_{m-1} is the energy mean wave period (s). Wave power was calculated in $\mathrm{W\,m^{-1}}$ and converted to $\mathrm{kW\,m^{-1}}$ by dividing by 1000.

This quantity was used as an index of temporal variation in regional offshore wave conditions. It does not represent wave-energy flux at the shoreline or the hydrodynamic force acting directly on individual carcasses because nearshore wave transformation, wave direction relative to the shoreline, local bathymetry, and wave breaking were not resolved by this analysis. Temporal variation in air temperature, relative humidity, tide level, offshore significant wave height, energy mean wave period, and estimated offshore wave power is presented in main-text Figure 2.

Summary statistics were calculated for the period from confirmation of death on July 31 to burial on August 7. Mean air temperature and relative humidity were calculated from the hourly JMA observations, whereas summary statistics for wave conditions were calculated from the two-hourly ERA5 records. During this period, mean air temperature and relative humidity were 28.7°C and 78%, respectively. Offshore significant wave heights mostly exceeded 1.2 m, and estimated offshore wave power was generally around 10 $\mathrm{kW\,m^{-1}}$ but briefly exceeded 40 $\mathrm{kW\,m^{-1}}$ during the early hours of August 2 (Day 2). No rainfall was recorded during the observation period. The carcasses were stranded between the mouths of two small rivers (Figure S1), but given the small size of these rivers and the absence of rainfall, river-derived currents were likely minor relative to wave action and tidal movement.

Chronology of carcass decomposition

July 31 [Day 0]
A: Code II→III, B: Code II→III, C: Code II→III, D: Code II→III
Max 33.3°C, Min 24.9°C; High tide 08:11, Low tide 14:04

On the morning of July 30, at least two of the whales were observed exhaling (Code I), but by noon on July 31, all four individuals were confirmed dead. None of the whales exhibited conspicuous external injuries or fishing gear–related damage. From July 29 to midday on July 31, the positions of the whales showed little change. At midday on July 31, the black coloration and wrinkled trunk surface characteristic of living sperm whales remained evident in A–D (Code II; Figure S2b, d). However, some individuals showed bleeding from the gums and oral cavity due to relaxation of the jaw joints, slightly tinting the surrounding seawater red (Figure S2a, c). By the evening of July 31, the four carcasses had gradually shifted northwest along the shore. D was separated from the closely clustered A–C (Figure S3a, b). Raised flippers were observed in several carcasses (Figure S3b, c), indicating the onset of trunk bloating (Code III) (Geraci and Lounsbury, 2005).

August 1 [Day 1]
A: Code III, B: Code III, C: Code III, D: Code III
Max 30.5°C, Min 25.0°C; High tide 09:23, Low tide 14:17

In the early afternoon of August 1, the spatial arrangement of the four carcasses remained unchanged from the evening of the previous day (Figure S4a, b). Raised flippers and penile protrusion were observed in all carcasses (Figure S4b–d), indicating continued accumulation of internal gases and progression of decomposition. In D, epidermal slippage was observed on parts of the body (Figure S4c), whereas A–C remained entirely covered by black epidermis.

August 2 [Day 2]
A: Code III, B: Code III, C: Code III, D: Code IV
Max 32.5°C, Min 25.5°C; High tide 11:18, Low tide 14:49

The spatial arrangement remained similar to that of the previous days, with D separated from clustered A–C (Figure S5a). Clear epidermal slippage was observed in A–D, accompanied by progressive discoloration of the body surface and blubber dissolution (Figure S5a–d). Decomposition stages differed among carcasses: in A–C, internal gas accumulation maintained raised flippers (Figure S5c, d), whereas in D, gas had escaped and the flippers had returned to a lowered position (Geraci and Lounsbury, 2005). Trunk rigidity was lost in D, and blubber decomposition had progressed further than in A–C (Figure S5b).

August 3 [Day 3]
A: Code IV, B: Code IV, C: Code IV, D: unknown
Max 33.5°C, Min 25.5°C; no distinct high or low tide was predicted

The overall positions of A–C remained unchanged, although inter-carcass distances and arrangements shifted slightly. Internal gases had escaped from the trunks, resulting in folded flippers (Figure S6a–c). Discoloration and blubber dissolution progressed throughout the body, while the overall three-dimensional trunk structure was maintained. Wave impacts induced active dorsoventral oscillations of the caudal region in A and B (chronological video), resulting in fraying of the flukes and loss of their original shape. In B, the lower jaw was exposed. In D, by contrast, only an exposed occipital bone was observed on the beach that day, whereas the mandibles, cranial soft tissues, and trunk were not found and were presumed to have drifted offshore (main-text Figure 1b; Figure S6d).

August 4 [Day 4]

A: Code IV, B: Code IV, C: Code IV, D: unknown

Max 32.4°C, Min 27.5°C; Low tide 07:06, High tide 16:12

A–C moved approximately 300–500 m northwest along the shore (Figure S1c), with A positioned about 100 m northwest of B and C. Complete epidermal slippage from the trunk surface was observed in A–C, exposing the white blubber layer, while trunk rigidity and a rounded trunk shape were retained (Figure S7a, b). The lateral flippers of A–C retained black epidermis. As on the previous day, the mandibles, cranial soft tissues, and trunk of individual D were not observed.

August 5 [Day 5]

A: Code IV, B: Code IV, C: Code IV, D: unknown

Max 32.4°C, Min 27.8°C; Low tide 08:09, High tide 16:04

B and C showed little positional change, whereas A moved southeast toward B and C. This was the only movement observed in a direction other than northwest during the observation period (Figure S1c). Around 08:00 during low tide, the scientific survey led by the Institute of Cetacean Research, Japan was conducted, during which body length measurements were permitted for the first time. Based on their stranding positions, the carcasses were designated A–C from northwest to southeast, and the detached cranial skeletal remains were assigned to individual D. These individual IDs were retrospectively applied to all earlier observations and photographs. The total body lengths of A–C were 14.0, 13.5, and 12.8 m, respectively.

All three carcasses present at the site (A–C) exhibited frayed flukes. Distinct neck constrictions were observed in A and B, exposing parts of the mandible and maxilla (Figure S8a–c; 3D datasets). In D, the maxilla (Figure S8e), vomer (Figure S8f), occipital bone (Figure S8g), and some viscera (Figure S8h) were observed, whereas the cranial soft tissues and the trunk were not identified. Upper jaw teeth and muscle fragments near the dorsal fin were legally collected from A–C for research purposes. Lower jaw teeth were already missing when the scientific survey began; however, given the large body size of the whales, this limited sampling was considered to have had a negligible effect on the present study.

During high tide, wave-induced dorsoventral oscillations of the caudal region and neck were observed in A–C, accompanied by trunk flotation and short-term offshore displacement, particularly in A and B (Figure S9; chronological video). At this stage, the skulls and mandibles of A–C remained attached to the trunks.

August 6 [Day 6]

A: Code IV, B: Code IV, C: Code IV, D: unknown

Max 36.5°C, Min 28.1°C; Low tide 08:49, High tide 16:36

During the morning low-tide survey, the overall positions of A–C remained largely unchanged; A moved closer to C, while B remained slightly separated. Complete skull and mandible detachment occurred in A and B, with scattered occipital bones near A (Figure S10b; chronological video). The detached maxilla near B was documented later the same day (main-text Figure 1c; Figure S11e). The mandibles were not found and were presumed to have drifted offshore. Cranial soft tissues, however, remained attached to the trunks. Some ribs and vertebrae were exposed (Figure S10a and S11a), although most skeletal elements remained covered by blubber, preserving the three-dimensional trunk shape. C retained cervical articulation with minimal soft tissue loss (Figures S10d and S11d). In D, scattered cranial bone elements were observed as on the previous day, while the trunk remained unconfirmed.

During high tide, wave action caused collapse of A's body cavity, resulting in repeated drifting and re-stranding (Figure S12a). In B, cracks developed in cranial soft tissues, causing asynchronous oscillation between the cranial soft-tissue mass and the trunk (Figure S12b–d; chronological video). C, positioned slightly uphill relative to A and B, showed no significant wave-induced flotation (Figure S12e).

August 7 [Day 7]

A: Code V, B: Code IV (or V), C: Code IV, D: Code V

Max 33.2°C, Min 27.6°C; Low tide 09:29, High tide 16:55

A and B drifted slightly northwest, likely due to repeated minor re-stranding events, whereas C remained nearly stationary (Figure S1c). During the morning low tide, A's body cavity had completely collapsed, and its overall external body form was no longer recognizable (Figure S13a). Vertebral articulations were partially preserved by residual fat and connective tissue, but their spatial arrangement was disrupted (chronological video). In B, blubber decomposition had progressed, but the three-dimensional trunk shape was largely retained (Figure S13b). Cranial soft tissues remained partially connected to the trunk. In C, a dorsal neck constriction was observed, although the head–trunk connection was maintained (Figure S13c). Flipper decomposition progressed slowly, with more than half of the epidermis remaining (Figure S13d).

In individual D, the trunk was found approximately 1 km northwest of the separated cranial skeletal elements, including the maxillae, vomer, and squamosal bone (Figure S13e–g; 3D datasets). Although the trunk cavity had collapsed, the trunk still exceeded 10 m in length and appeared to retain the cranial soft tissues (Figure S13h; 3D datasets). Distinct ellipsoid structures characteristic of cranial soft tissues (Okabe et al., 2021; Figure S13i) suggested that the cranial soft tissues and trunk had remained connected.

Subsequently, the Awa Public Works Office buried the large carcass remains and recoverable bones using excavators (Figure S14). Scattered bones could not be fully recovered, and some remained in situ. Small cranial elements, including the ear bones, were not recovered; therefore, their disarticulation or breakage could not be directly assessed.

References

Copernicus Climate Change Service, Climate Data Store. 2023. ERA5 hourly data on single levels from 1940 to present. Copernicus Climate Change Service Climate Data Store. https://doi.org/10.24381/cds.adbb2d47.

Geraci, J. R., and Lounsbury, V. J. 2005. *Marine mammals ashore: A field guide for strandings.* Second edition. National Aquarium in Baltimore, Maryland. 371 pp.

Guillou, N. 2020. Estimating wave energy flux from significant wave height and peak period. *Renewable Energy* 155: 1383–1393. https://doi.org/10.1016/j.renene.2020.03.124.

Japan Meteorological Agency. 2025a. Tables of monthly climate statistics. https://www.data.jma.go.jp/stats/data/en/smp/index. Accessed 10 October 2025.

Japan Meteorological Agency. 2025b. Tidal observation data. https://www.data.jma.go.jp/kaiyou/db/tide/genbo/index. Accessed 10 October 2025.

Katada, Haruka. 2026. Wave-driven rapid detachment of the skull in stranded sperm whales. figshare. Media. https://doi.org/10.6084/m9.figshare.31145917.v1

Katooka, D., Sakiyama, T., Isikawa, H., Yamada, T. K., Tajima, Y., and Taru, H. 2020. Review of sighting, stranding, and bycatch reports of baleen whales along the coast of Sagami Bay. *Natural History Report of Kanagawa* 41: 83–93. https://doi.org/10.32225/nkpmnh.2020.41_83.

Okabe, H., Karino, H., Tamaki, Y., Ozawa, S., and Kobayashi, N. 2021. Stranded sperm whale at Naha Airport: the process of decomposition and disposal of a whale carcass. *Proceedings of the 2021 Okinawan Block Conference of Land, Infrastructure and Transport*. [Japanese]

Ramos, H. G. da C., Colosio, A. C., Marcondes, M. C. C., Lopez, R. P. G., Michalski, B. E., Ghisolfi, R. D., Gonçalves, M. I. C., and Bovendorp, R. S. 2024. Postmortem interval applied to cetacean carcasses: observations from laboratory and field studies with the Abrolhos Bank Region, Brazil. *Forensic Science International: Animals and Environments* 5: 100082. https://doi.org/10.1016/j.fsiae.2024.100082.

Sumi, M., Hanaue, R., Sakiyama, T., Suzuki, S., Ishikawa, H., Yamada, T. K., Tajima, Y., and Taru, H. 2022. Review of sighting, stranding, and bycatch of Odontoceti (Physeteridae, Kogiidae, Ziphiidae, and Phocoenidae) along the coasts of Sagami and Tokyo Bays. *Natural History Report of Kanagawa* 43: 1–23. https://doi.org/10.32225/nkpmnh.2022.43_1.

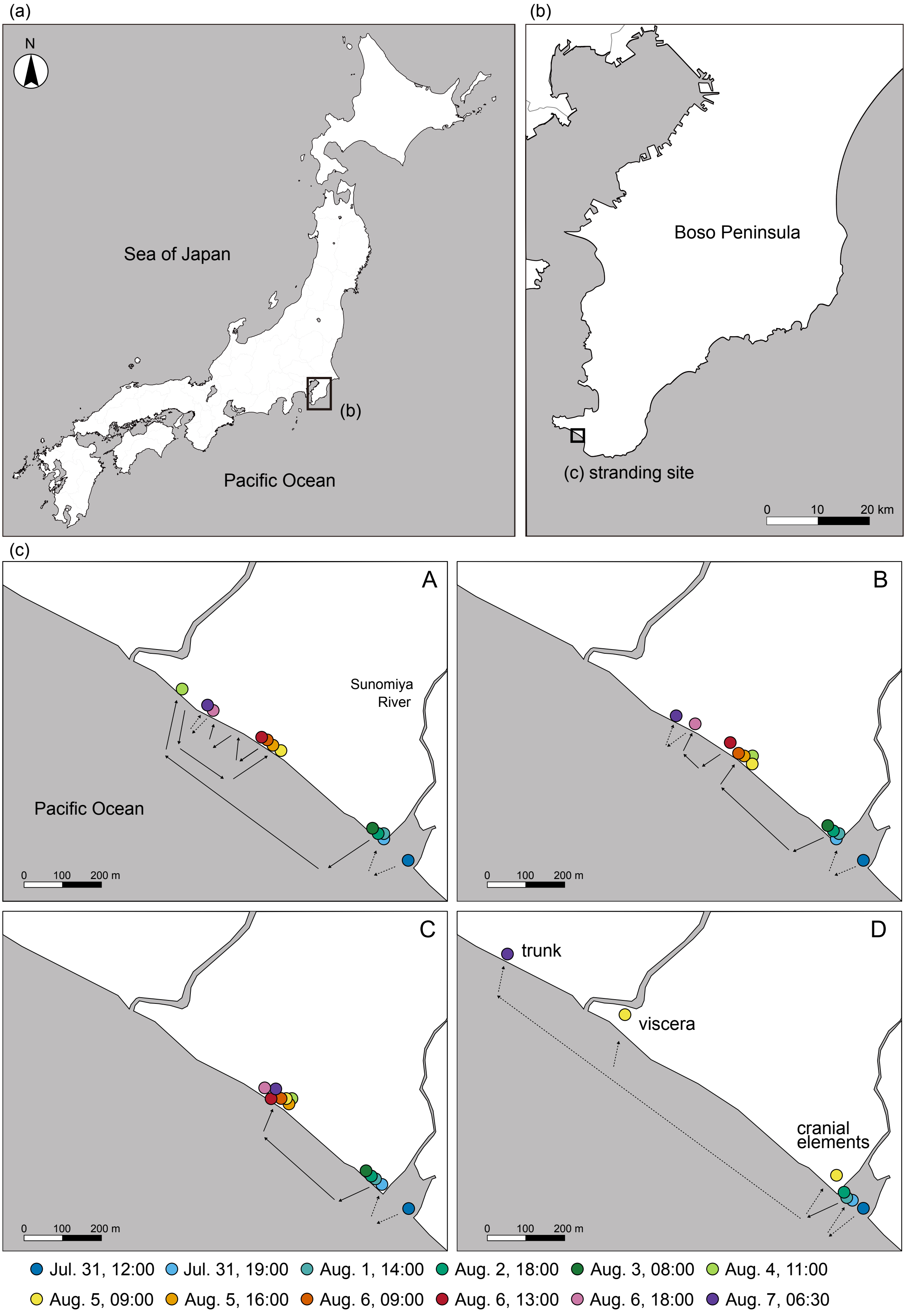

Figure S1 Map of the mass stranding site of four sperm whales. (a) Index map of Japan showing the location of the Boso Peninsula (b). The stranding site (Heisaura Beach, Tateyama City, Chiba, Japan) is marked by a square in (b), corresponding to the area shown in (c). (c) Positions of the carcasses and detached remains assigned to individuals A–D along Heisaura Beach from July 31 (Day 0) to August 7 (Day 7). Solid lines represent observed movements, and dashed lines represent estimated movements.

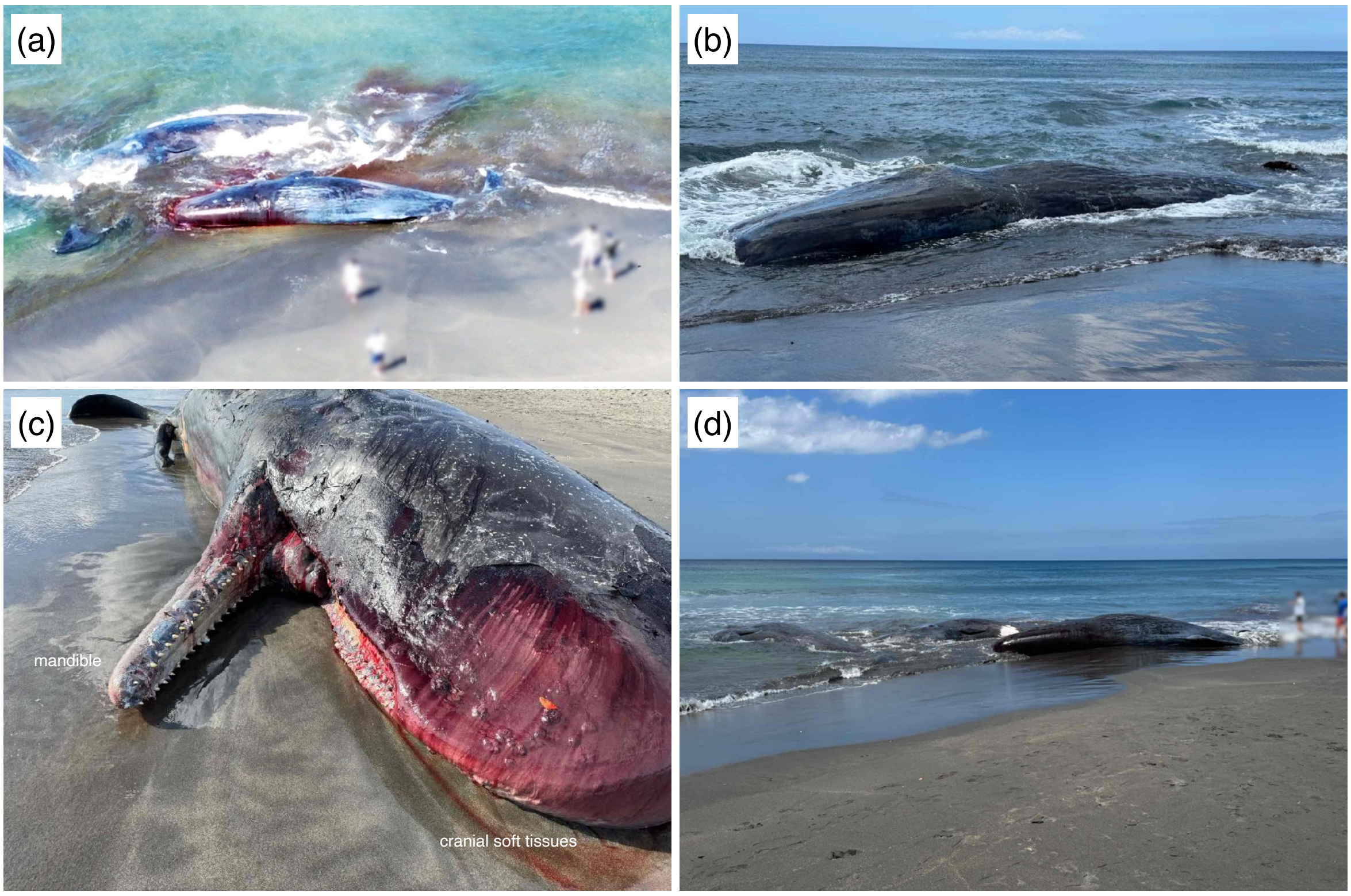

Figure S2 Stranded sperm whales on July 31 (Day 0) at 12:00 JST. (a) Overview of the stranding site. (b–d) Close-up views of selected individuals. All photographs by the Awa Public Works Office. People visible in the photographs were anonymized by pixelation.

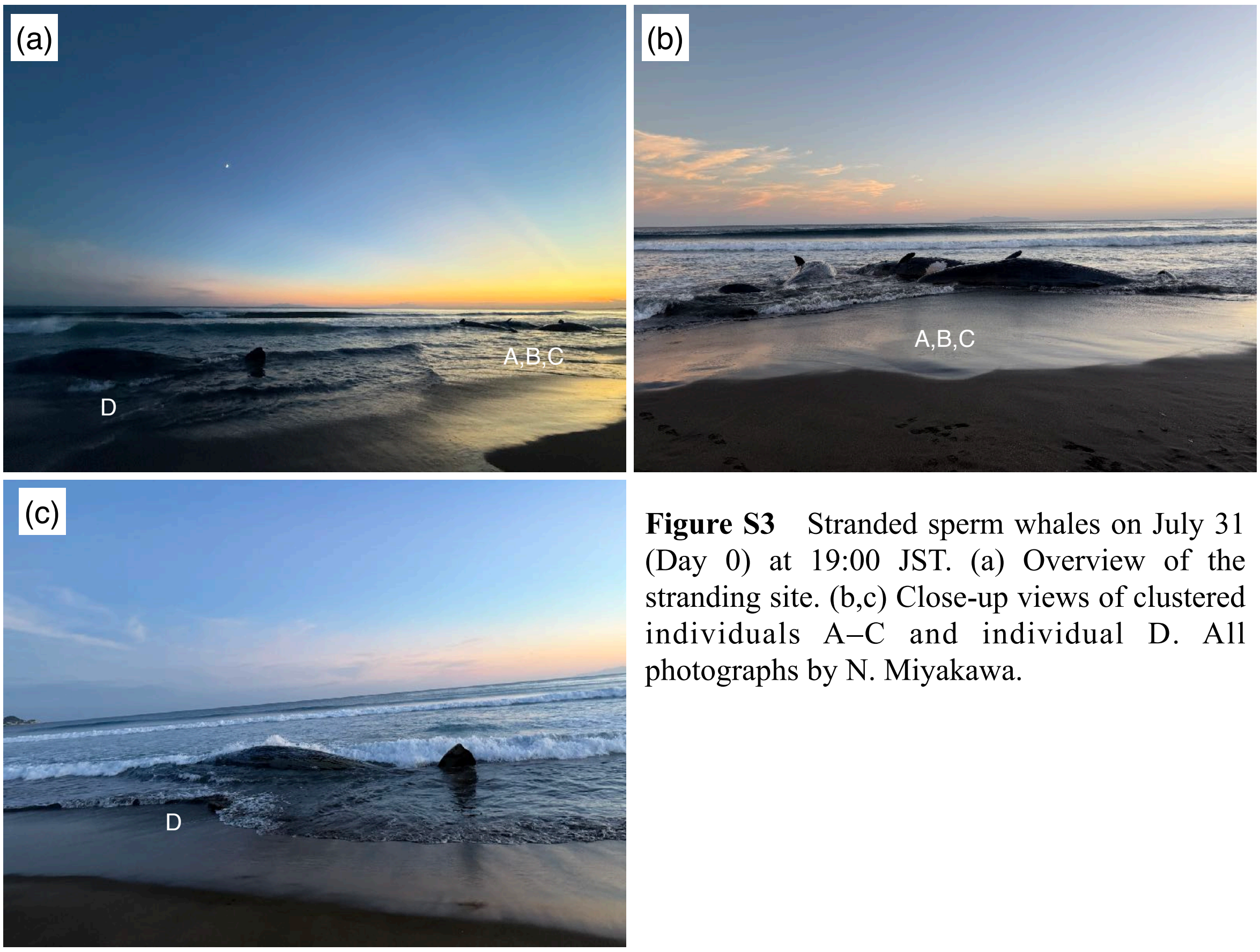

Figure S3 Stranded sperm whales on July 31 (Day 0) at 19:00 JST. (a) Overview of the stranding site. (b,c) Close-up views of clustered individuals A–C and individual D. All photographs by N. Miyakawa.

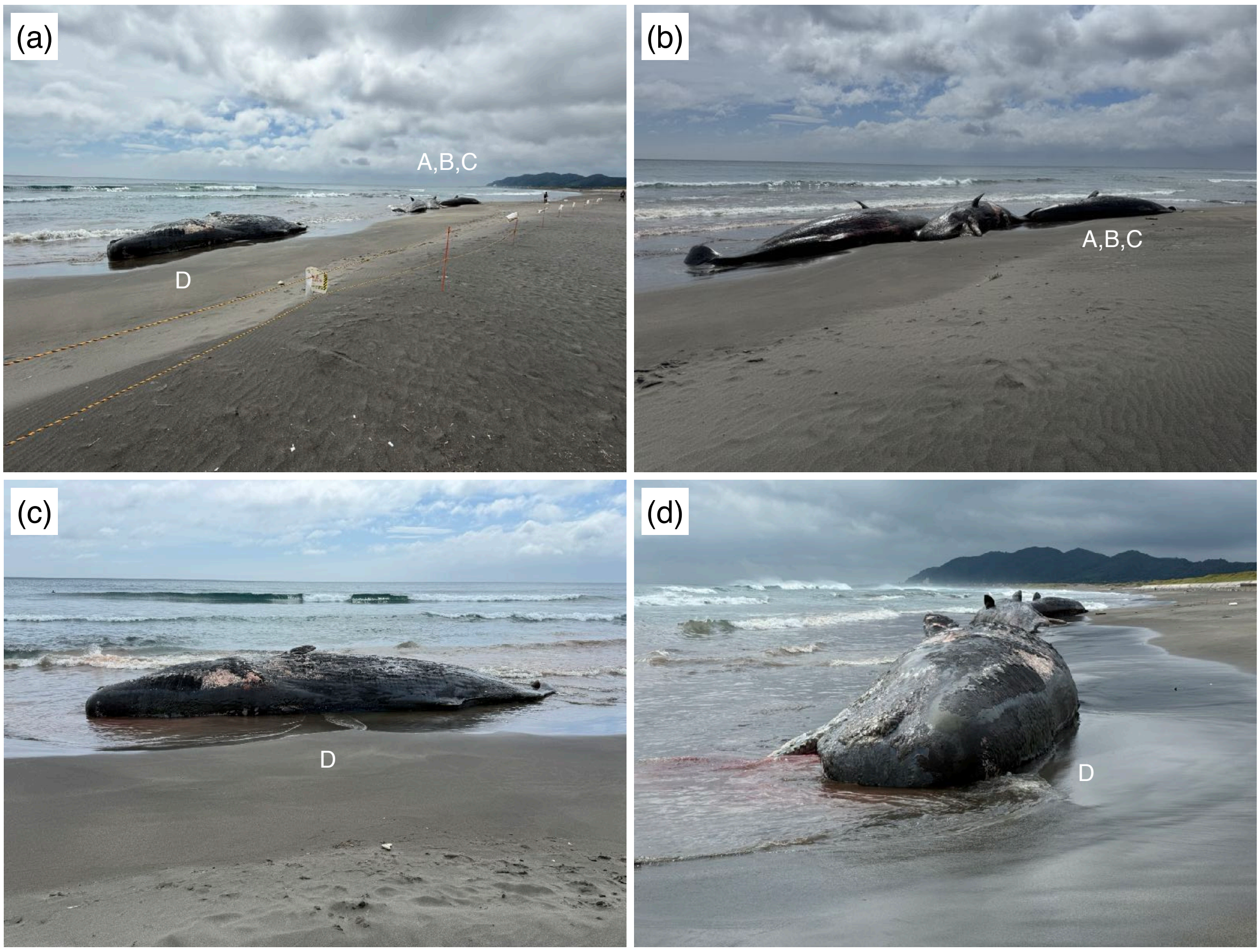

Figure S4 Stranded sperm whales on August 1 (Day 1) at 14:00 JST. (a) Overview of the stranding site. (b–d) Views of individual D and the A–C cluster. Photographs (a,b,d) by the Tateyama Marine Industries Office and (c) by the Awa Public Works Office.

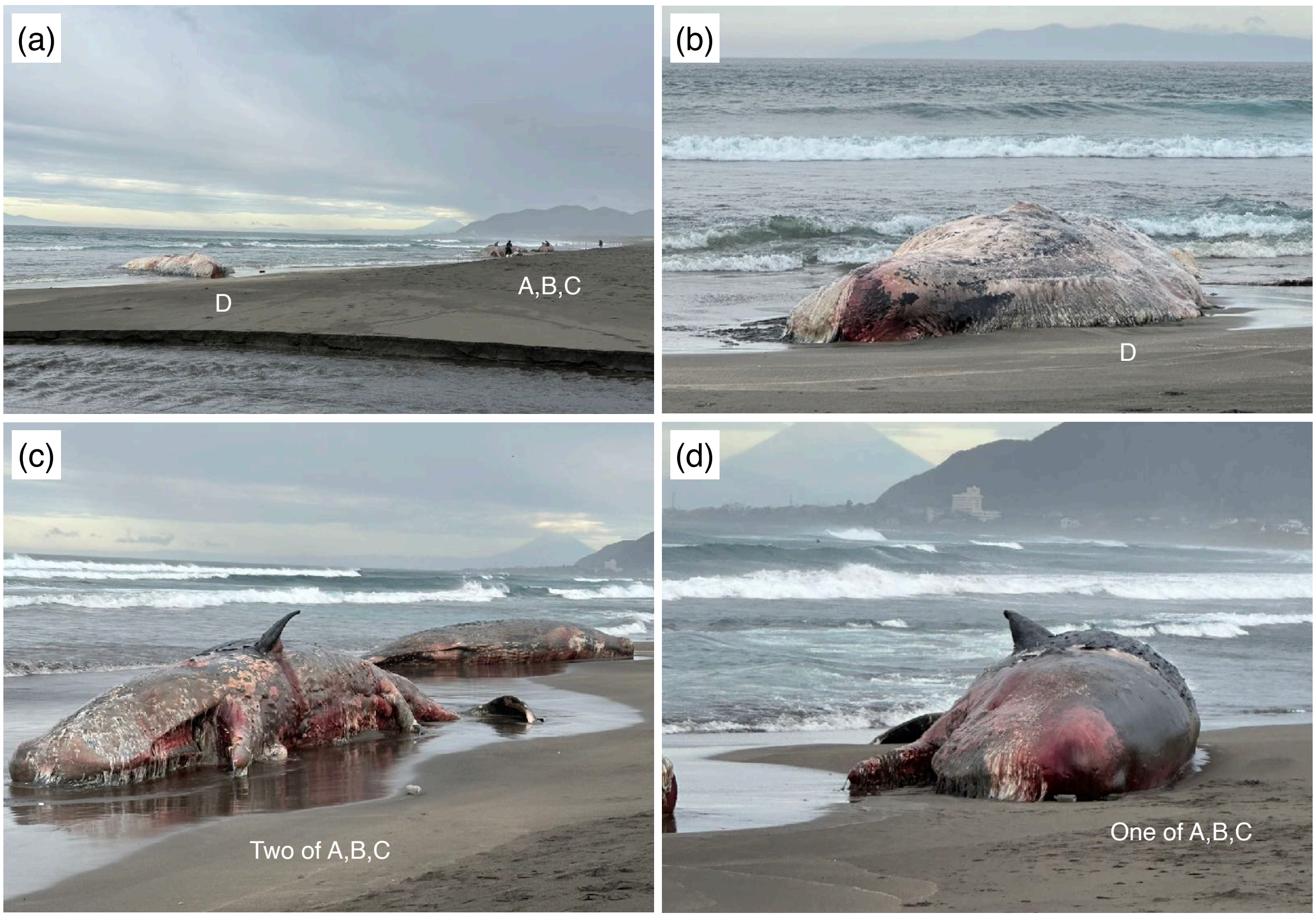

Figure S5 Stranded sperm whales on August 2 (Day 2) at 18:00 JST. (a) Overview of the stranding site. (b–d) Close-up views of individual D and selected individuals within the A–C cluster. All photographs by the Awa Public Works Office.

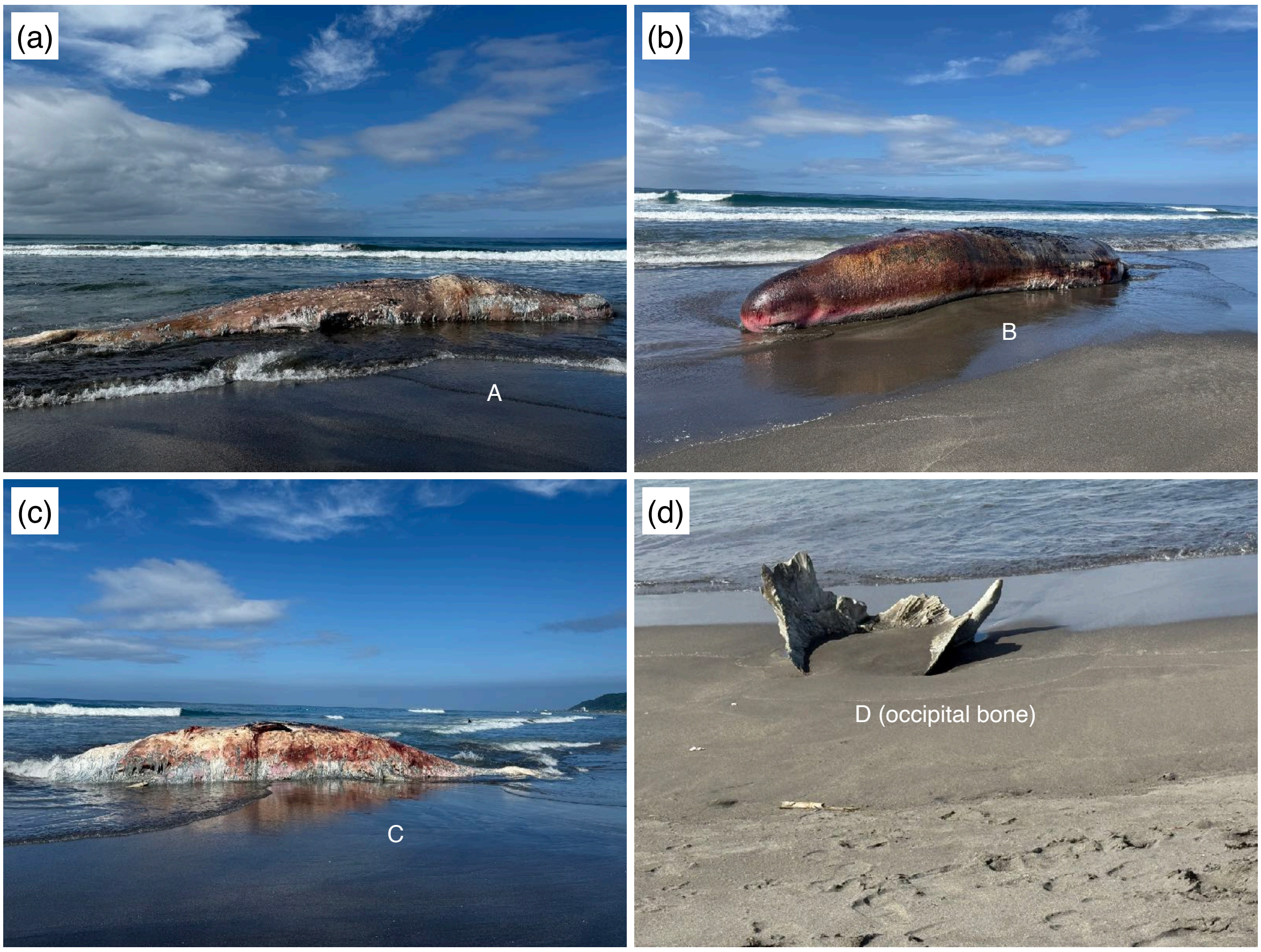

Figure S6 Stranded sperm whales on August 3 (Day 3) at 08:00 JST. (a–c) Close-up views of individuals A–C. (d) Detached occipital bone assigned to individual D. All photographs by N. Miyakawa.

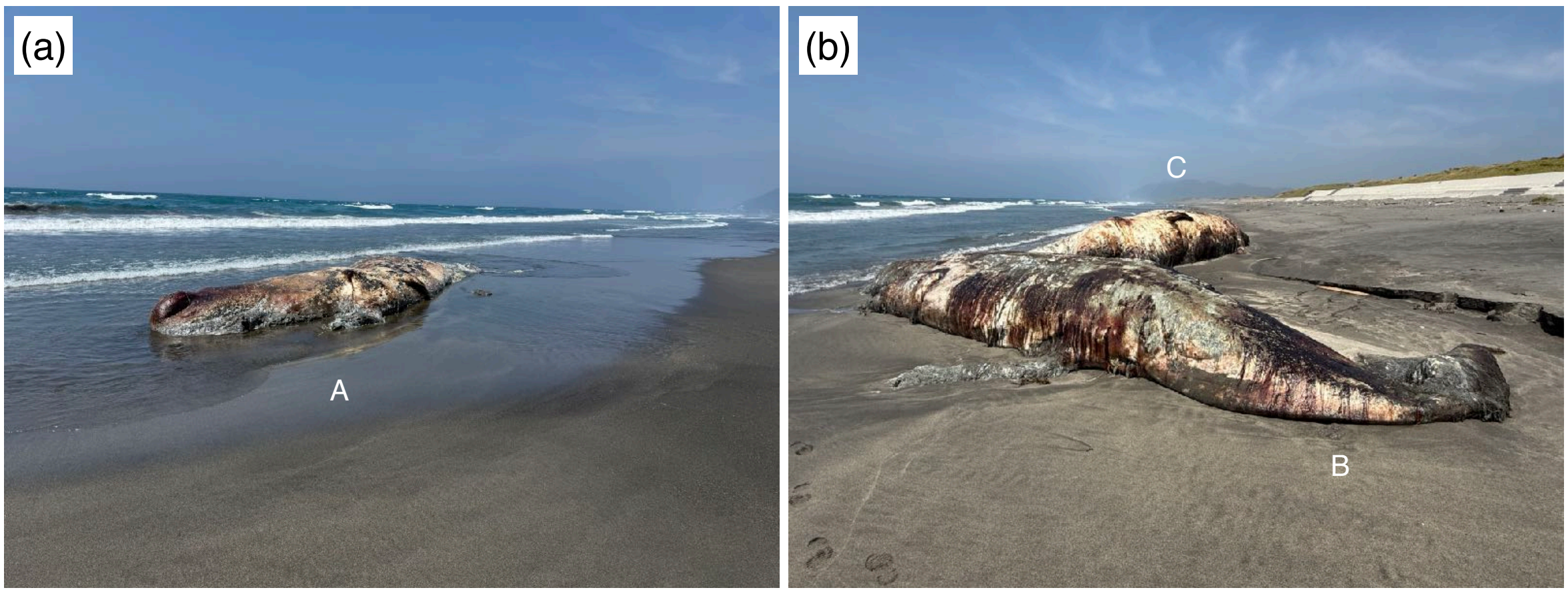

Figure S7 Stranded sperm whales on August 4 (Day 4) at 11:00 JST. (a,b) Close-up views of individuals A–C. All photographs by the Tateyama Marine Industries Office.

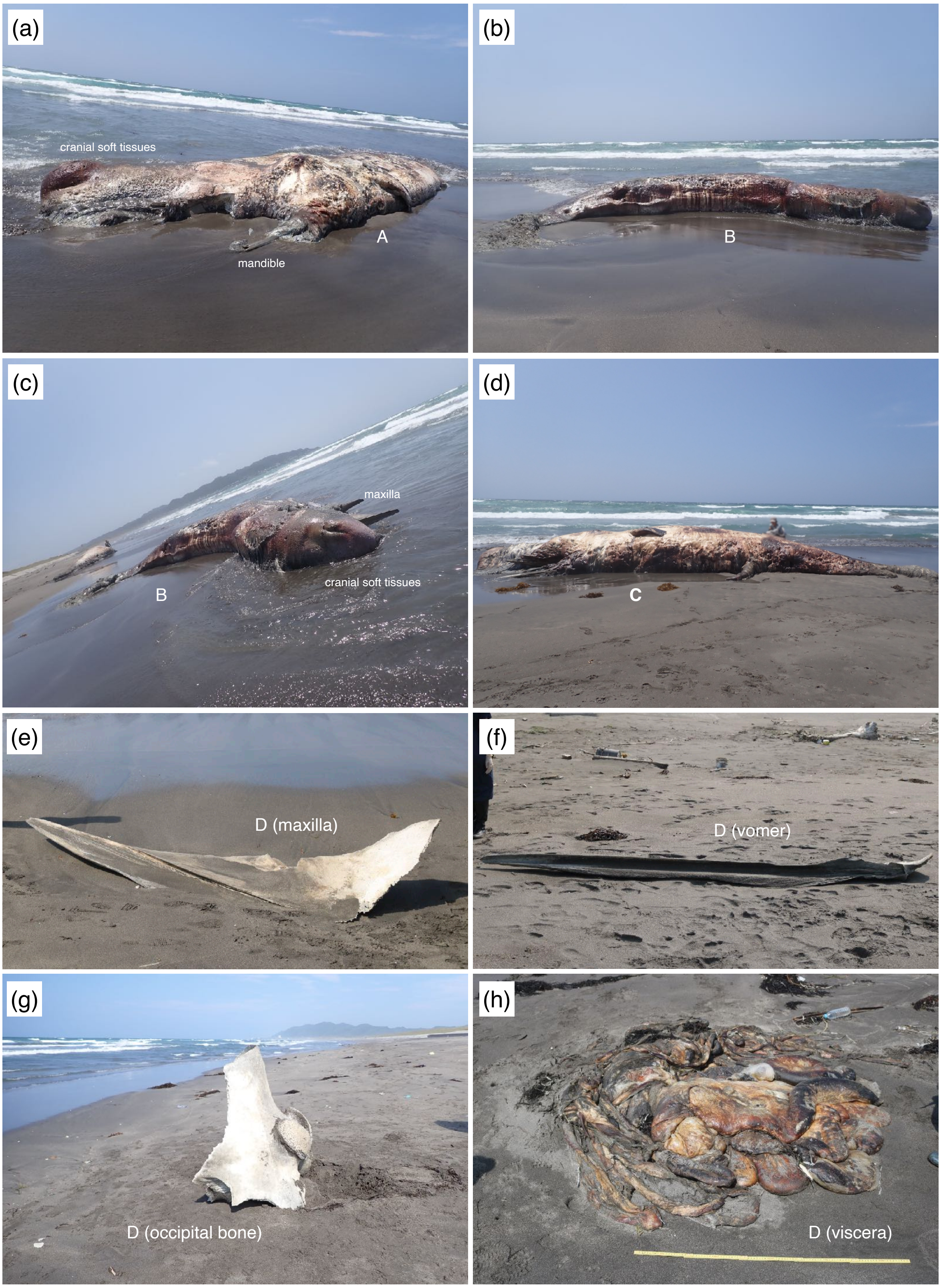

Figure S8 Stranded sperm whales on August 5 (Day 5) at 09:00 JST. (a–h) Close-up views of individuals A–C and detached skeletal elements and viscera assigned to individual D. Individual IDs are shown in each photograph. Photographs (a–d,g) by N. Mitsuoka and (e,f,h) by H. Katada. People visible in the photographs were anonymized by pixelation.

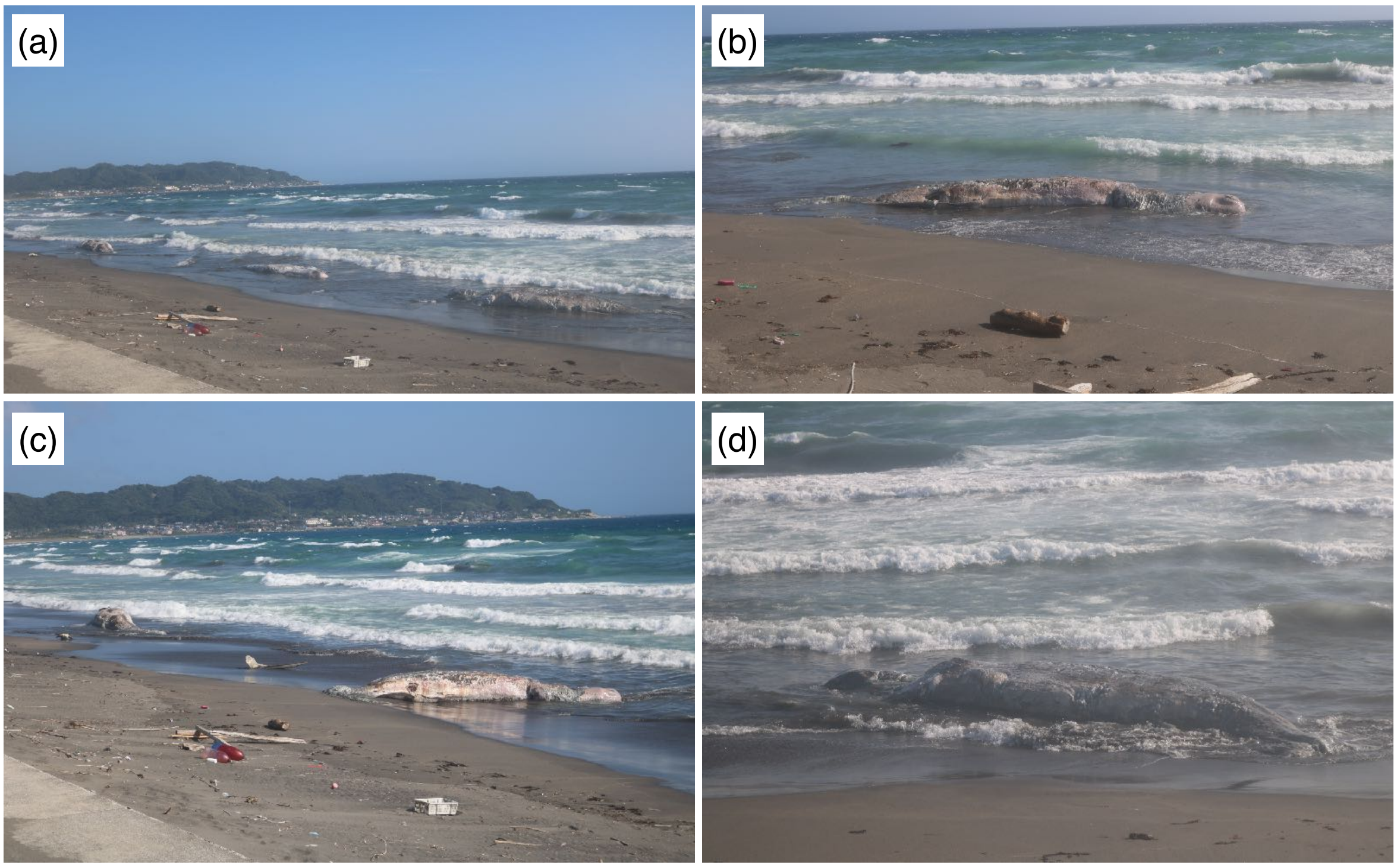

Figure S9 Stranded sperm whales on August 5 (Day 5) at 16:00 JST. (a) Overview of the stranding site. (b–d) Close-up views of the stranded carcasses during high tide. All photographs by H. Katada.

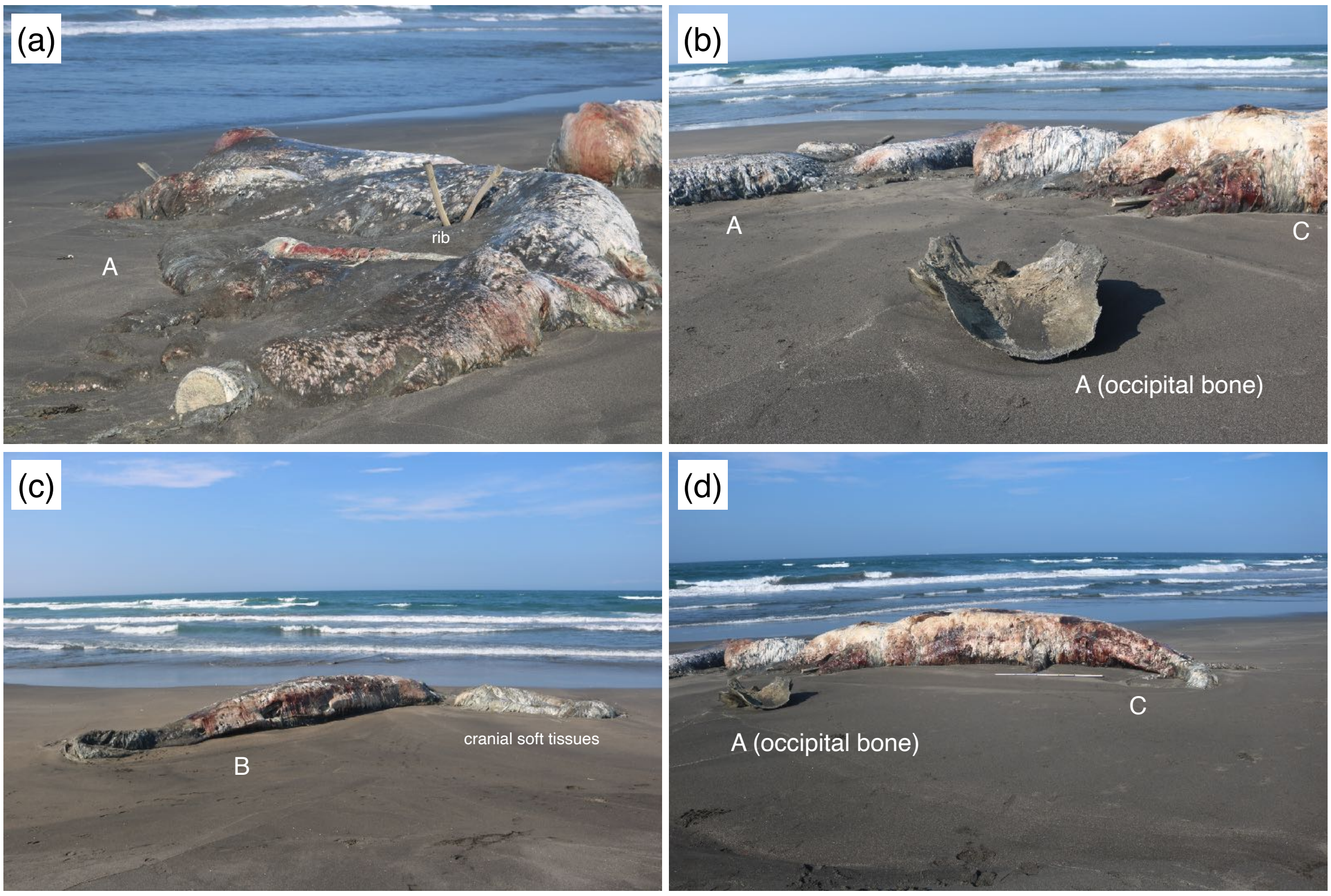

Figure S10 Stranded sperm whales on August 6 (Day 6) at 09:00 JST. (a–d) Close-up views of individuals A–C and the detached occipital bone near individual A. Individual IDs are shown in each photograph. All photographs by H. Katada.

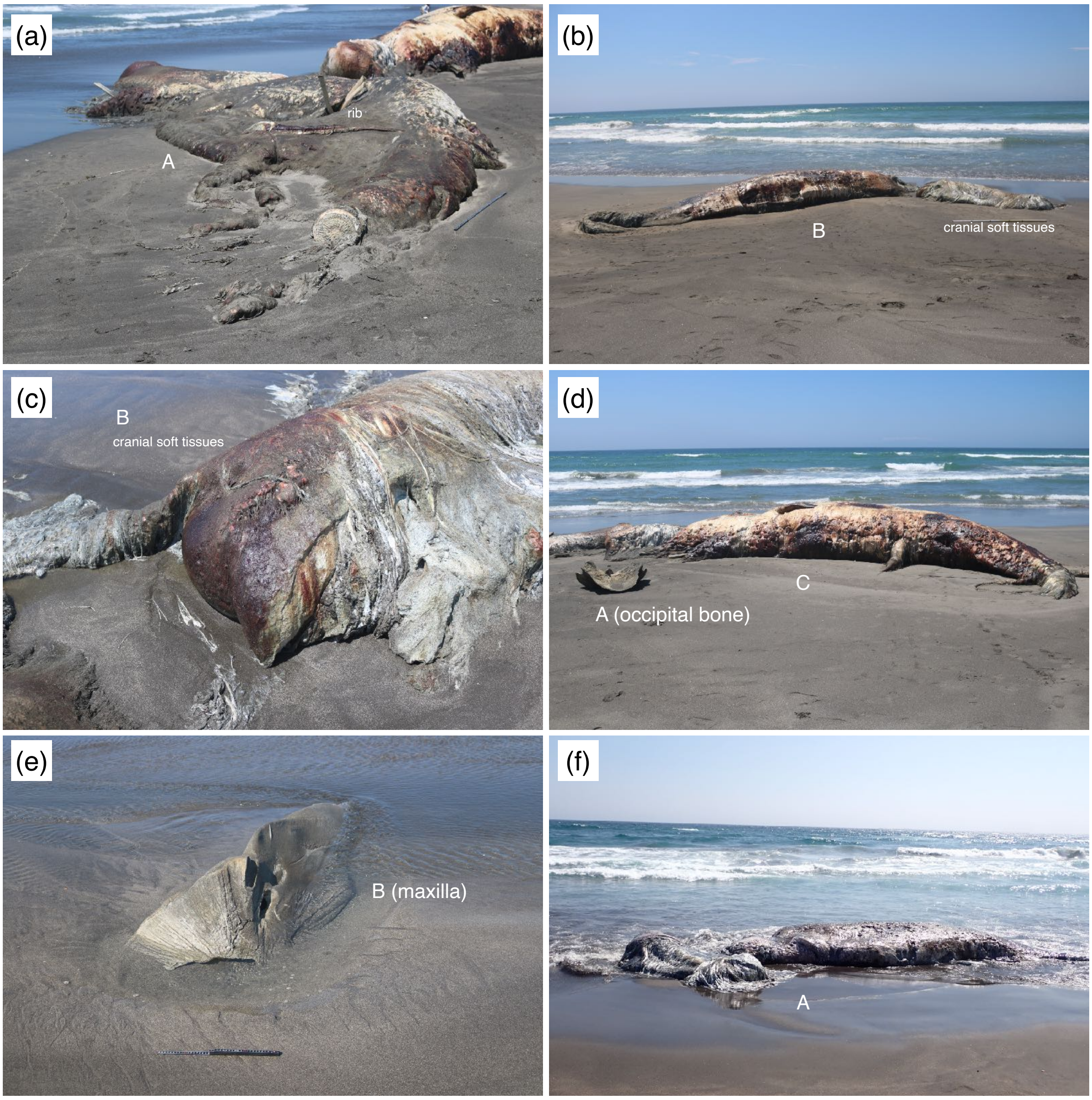

Figure S11 Stranded sperm whales on August 6 (Day 6) at 13:00 JST (a–e) and 14:00 JST (f). Close-up views of individuals A–C and detached cranial skeletal elements. Individual IDs are shown in each photograph. Photographs (a–e) by H. Katada and (f) by N. Mitsuoka.

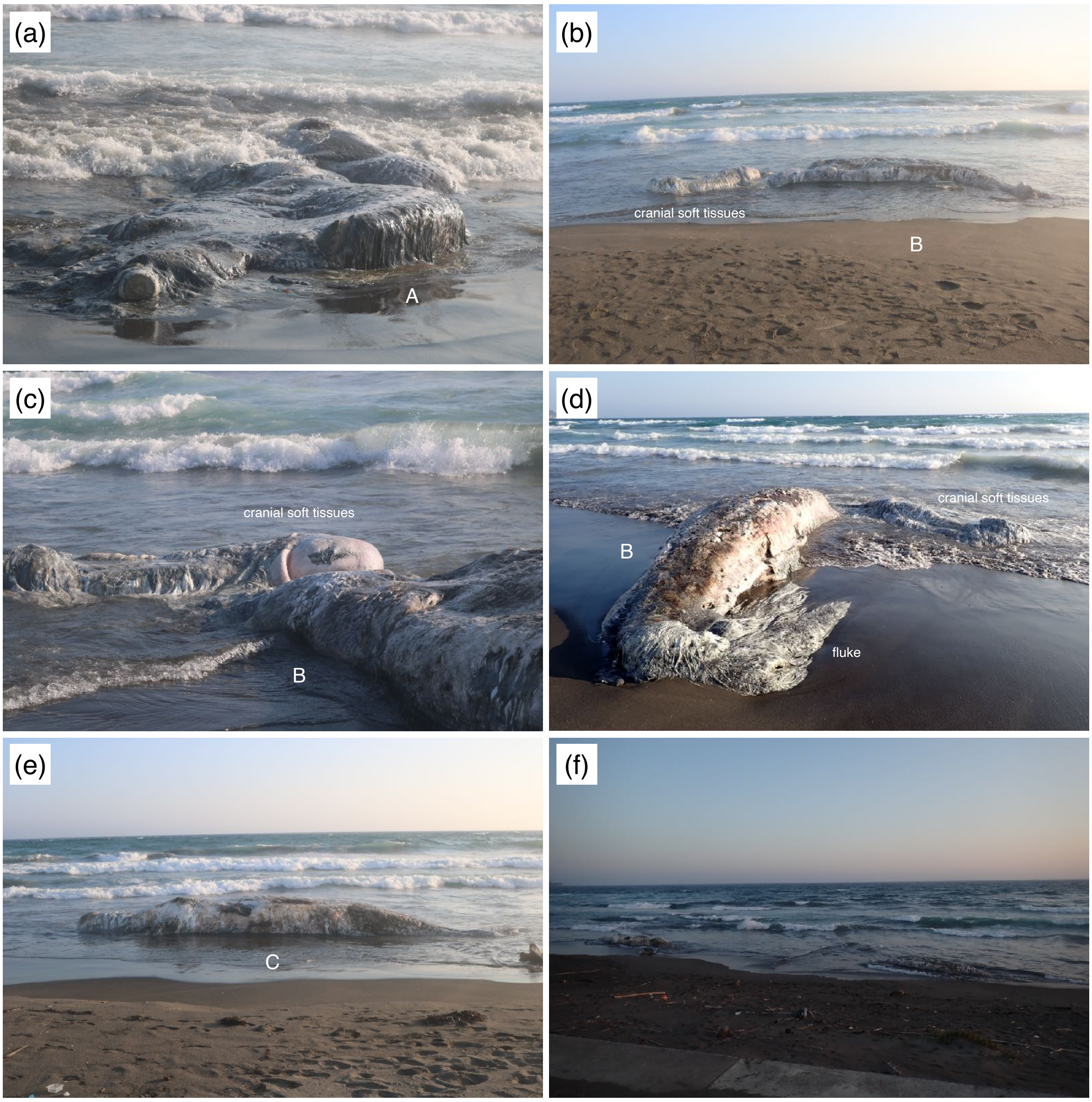

Figure S12 Stranded sperm whales on August 6 (Day 6) at 17:00 JST (a–e) and 18:00 JST (f). High-tide conditions showing collapse and flotation of individual A, deformation of the cranial soft tissues of B, and the relatively stable position of C. Photographs (a–c,e) by H. Katada and (d,f) by N. Mitsuoka.

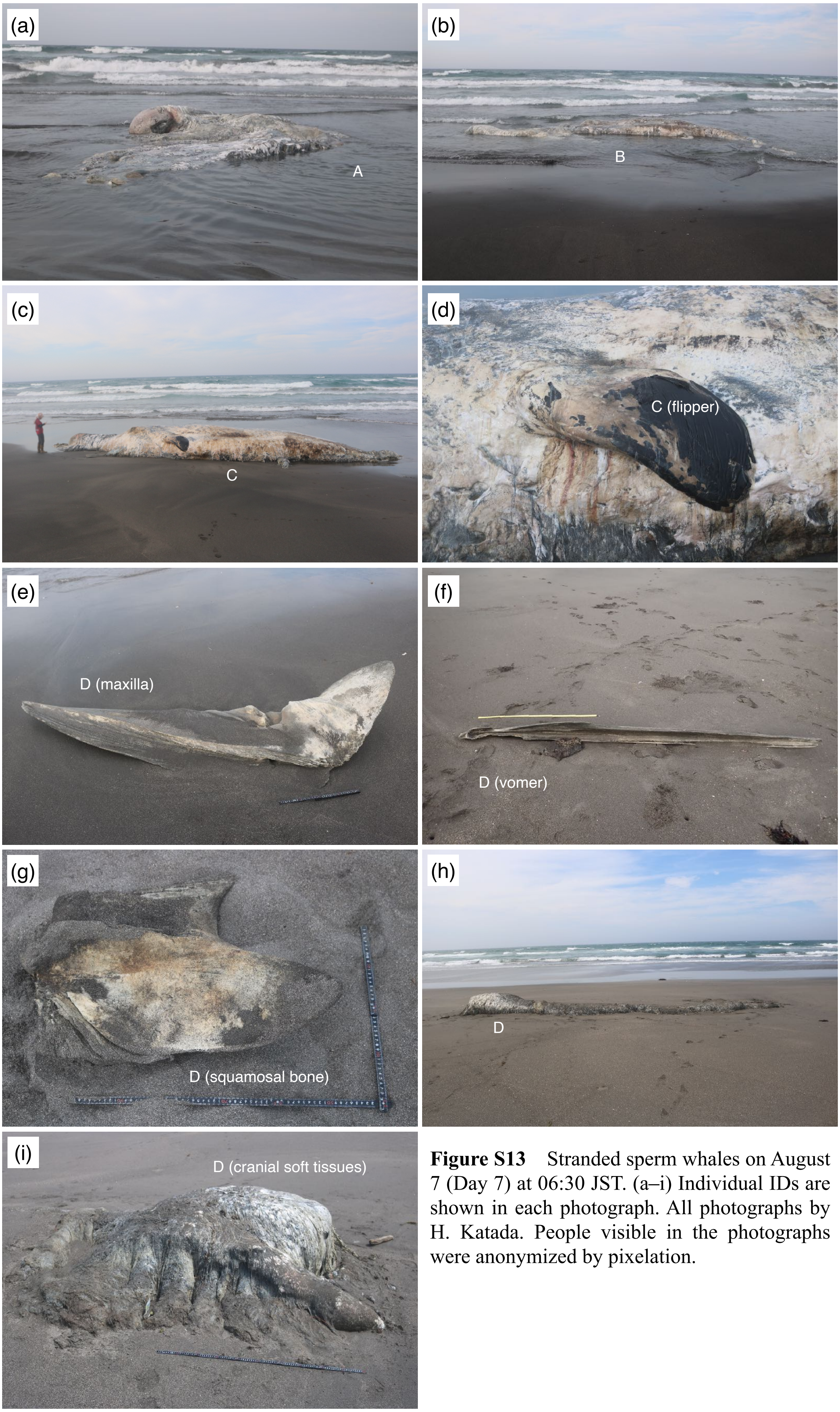

Figure S13 Stranded sperm whales on August 7 (Day 7) at 06:30 JST. (a–i) Individual IDs are shown in each photograph. All photographs by H. Katada. People visible in the photographs were anonymized by pixelation.

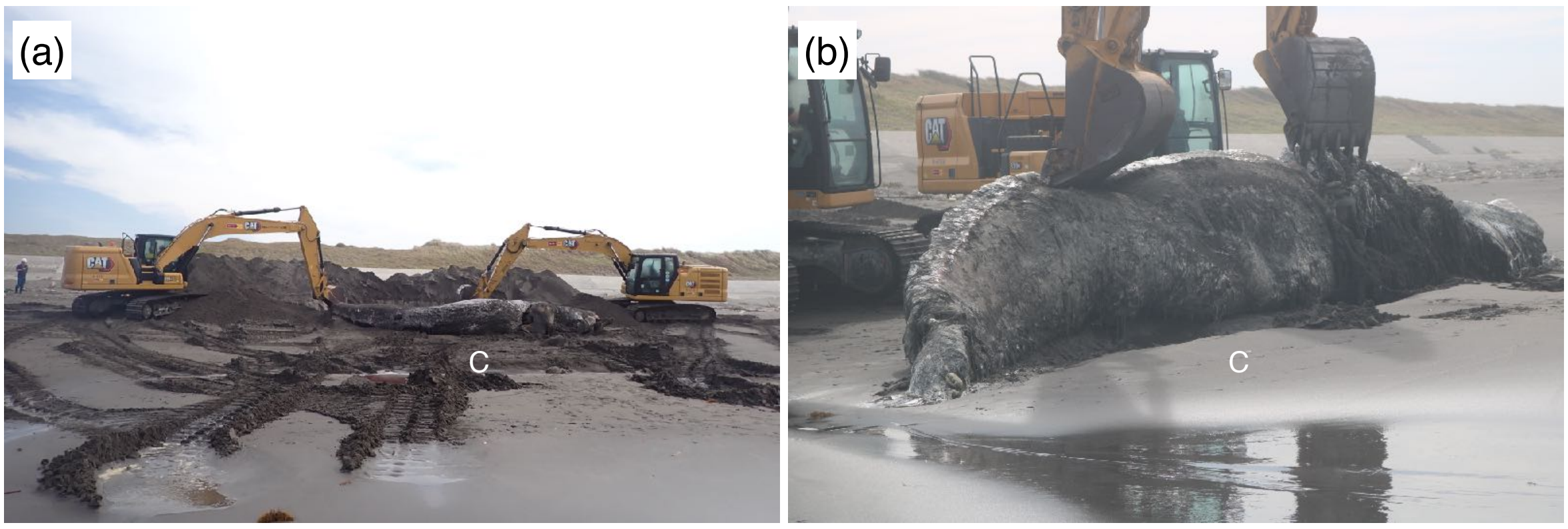

Figure S14 Burial operations on August 7 (Day 7) at 10:30 JST, showing individual C being buried using excavators. Photograph (a) by N. Mitsuoka and (b) by H. Katada.
